# Conformational changes of the phenyl and naphthyl isocyanate-DNA adducts during DNA replication and by minor groove binding molecules

**DOI:** 10.1093/nar/gkt608

**Published:** 2013-07-19

**Authors:** Shu-ichi Nakano, Yuuki Uotani, Yuichi Sato, Hirohito Oka, Masayuki Fujii, Naoki Sugimoto

**Affiliations:** ^1^Department of Nanobiochemistry, Faculty of Frontiers of Innovative Research in Science and Technology (FIRST), Konan University, 7-1-20, Minatojima-minamimachi, Chuo-ku, Kobe, 650-0047, Japan, ^2^Frontier Institute for Biomolecular Engineering Research (FIBER), Konan University, 7-1-20, Minatojima-minamimachi, Chuo-ku, Kobe, 650-0047, Japan, ^3^Department of Chemistry, Faculty of Science and Engineering, Konan University, 8-9-1, Okamoto, Higashinada-ku, Kobe, 658-8501, Japan, ^4^Molecular Engineering Institute (MEI), Kinki University, 11-6 Kayanomori, Iizuka, Fukuoka, 820-8555, Japan and ^5^Department of Environmental and Biological Chemistry, Kinki University, 11-6 Kayanomori, Iizuka, Fukuoka, 820-8555, Japan

## Abstract

DNA lesions produced by aromatic isocyanates have an extra bulky group on the nucleotide bases, with the capability of forming stacking interaction within a DNA helix. In this work, we investigated the conformation of the 2′-deoxyadenosine and 2′-deoxycytidine derivatives tethering a phenyl or naphthyl group, introduced in a DNA duplex. The chemical modification experiments using KMnO_4_ and 1-cyclohexyl-3 -(2-morpholinoethyl) carbodiimide metho-*p*-toluenesulfonate have shown that the 2′-deoxycytidine lesions form the base pair with guanine while the 2′-deoxyadenosine lesions have less ability of forming the base pair with thymine in solution. Nevertheless, the kinetic analysis shows that these DNA lesions are compatible with DNA ligase and DNA polymerase reactions, as much as natural DNA bases. We suggest that the adduct lesions have a capability of adopting dual conformations, depending on the difference in their interaction energies between stacking of the attached aromatic group and base pairing through hydrogen bonds. It is also presented that the attached aromatic groups change their orientation by interacting with the minor groove binding netropsin, distamycin and synthetic polyamide. The nucleotide derivatives would be useful for enhancing the phenotypic diversity of DNA molecules and for exploring new non-natural nucleotides.

## INTRODUCTION

Understanding the influences of adduct formation on DNA bases is important for the assessment of genetic toxicity, caused by the exposure to chemically active compounds and toxic substances. Bulky DNA adducts have biological interest in their carcinogenic activities and repair processes. The carbamoylated DNA lesions are produced by exposure to the isocyanate or isothiocyanate compounds that may be contained in the air and foods. It is reported that the methyl and aromatic isocyanates acting as electrophilic agents react with the exocyclic amino group of DNA bases and produce the carbamoylated products, most of the 2′-deoxycytidine adducts and few 2′-deoxyadenosine adducts (1). These adduct lesions affect the chromosome structure but appear to have little or no ability to induce gene mutations in a cell (2–4). However, the molecular basis of DNA replication across the bulky adduct lesions is poorly understood.

**Figure 1.**
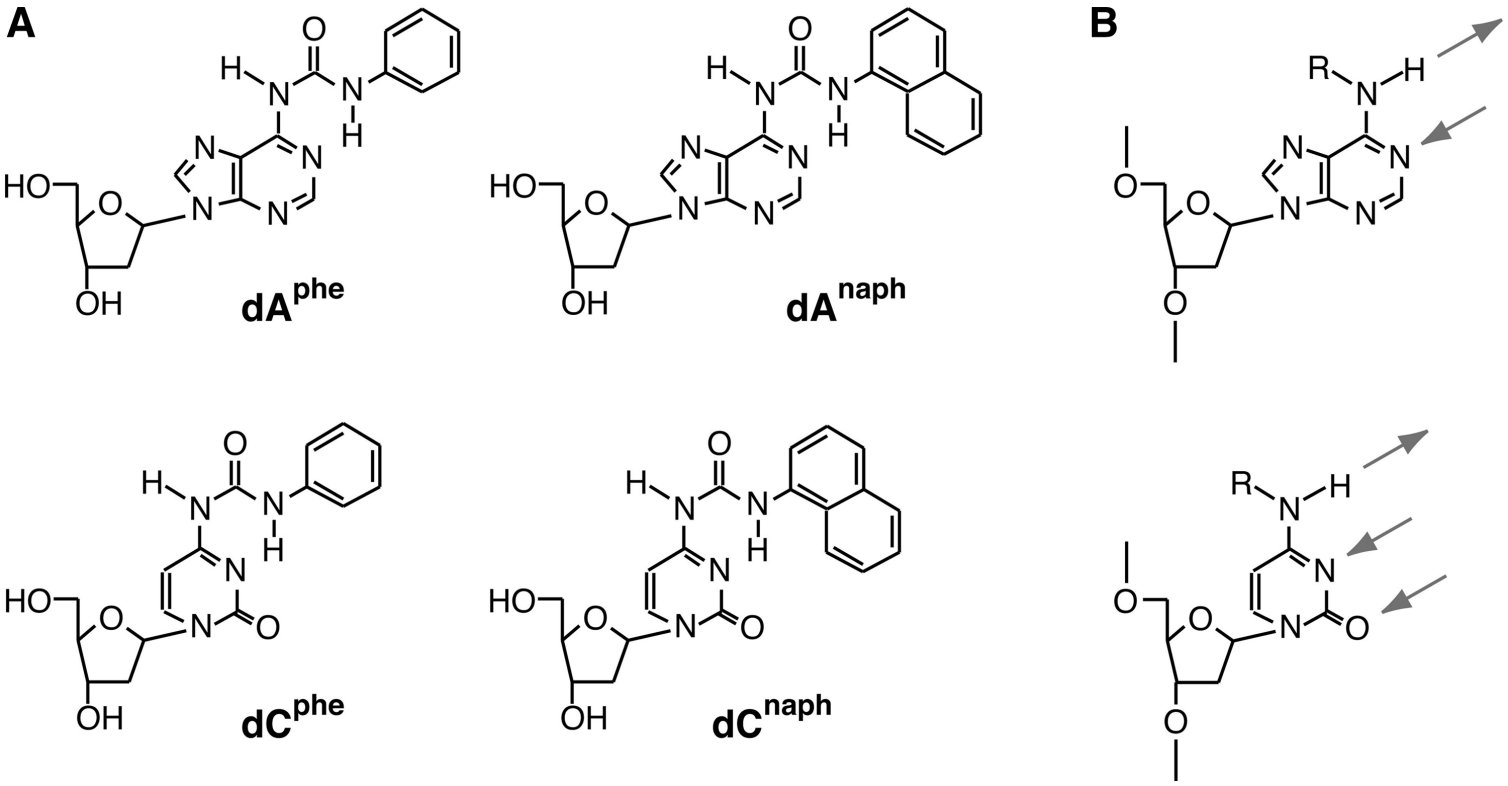
(**A**) Chemical structures of the 2′-deoxyadenosine and 2′-deoxycytidine derivatives used in this study. (**B**) The hydrogen bond acceptors and donor sites for Watson–Crick base pairing with thymine or guanine are indicated by arrows. R represents a phenylcarbamoyl or naphthylcarbamoyl group.

The DNA lesions produced by aromatic isocyanates have a bulky group, resulting in possessing two moieties with stacking properties, a nucleotide base and an attached aromatic group. As the attached aromatic group has the capability of a strong stacking interaction within a DNA helix, the adducts could affect the ability of base pairing. Figure 1A shows the carbamoylated DNA residues used in this study. An aromatic hydrocarbon group tethers to the amino groups of 2′-deoxyadenosine or 2′-deoxycytidine bases: the 2′-deoxyadenosine derivatives with a phenyl group [dA^phe^, *N*6-(*N*′-phenylcarbamoyl)-2′-deoxyadenosine] or a naphthyl group [dA^naph^, *N*6-(*N*′-naphthylcarbamoyl)-2′-deoxyadenosine] and the 2′-deoxycytidine derivatives with a phenyl group [dC^phe^, *N*6-(*N*′-phenylcarbamoyl)-2′-deoxycytidine] or a naphthyl group [dC^naph^, *N*6-(*N*′-naphthylcarbamoyl)-2′-deoxycytidine]. The phenyl and naphthyl groups are the bulky stacking groups, and their nucleotide base portions retain the hydrogen bonding sites for Watson–Crick base pairing (Figure 1B). One of the remarkable features of the adduct lesions is the possession of rotatable bonds that change the orientation of the phenyl or naphthyl group. These non-polar aromatic groups can stack in a nucleic acid duplex when the attached group occupies the Watson–Crick face of the adenine or cytosine moiety. In contrast, base pairing with a complementary thymine or guanine through hydrogen bonding is allowed when the phenyl or naphthyl group is positioned in an extrahelical conformation (5). In this study, the chemical modification and polymerase and ligase experiments were examined. We show that the bulky lesions are compatible with the DNA ligase and DNA polymerase reactions, although the adducts having a high-stacking propensity do not form a base pair with their complementary bases in solution. The conformation of the aromatic adducts changes depending on their interaction energies and can be modulated using minor groove binding molecules. The results would be useful for exploring new non-natural nucleotides for use in molecular biology studies and for developing nucleic acid drugs.

## MATERIALS AND METHODS

### DNA preparation

The 2′-deoxyadenosine and 2′-deoxycytidine derivatives shown in Figure 1A were synthesized and identified as previously described (6,7). DNA oligonucleotides containing the nucleotide derivatives and those labeled with Cyanine 3 (Cy3) at their 5′-ends were synthesized on a solid support using conventional phosphoramidite chemistry with an automated DNA synthesizer (Model 391, Applied Biosystems). The oligonucleotides were purified by reverse-phase high-performance liquid chromatography (HPLC) with a C18 column or by 20% polyacrylamide gel electrophoresis (PAGE) containing 7 M urea after removing the protective groups of the DNA bases. Their molecular weights were determined by matrix-assisted laser desorption/ionization time of flight mass spectroscopy (Voyager, PE Biosystems). DNA oligonucleotides labeled with 6-carboxyfluorescein (FAM) at their 5′-ends and natural DNAs of HPLC purification grade were purchased from Hokkaido System Science.

### DNA chemical modifications

The chemical modifications were conducted using freshly prepared KMnO_4_ and 1-cyclohexyl-3 -(2-morpholinoethyl) carbodiimide metho-*p*-toluenesulfonate (CMCT). The DNA duplexes used for the chemical modifications were prepared by mixing a fluorescently labeled sequence with a non-labeled complementary sequence. The oligonucleotide strand or structure concentration of 40 µM in a buffer containing 50 mM Tris–HCl (pH 7.5) and 10 mM MgCl_2_ were annealed by heating to 90°C before the modifications. The reagents used for the experiments were purchased from Wako, except for tRNA (phenylalanine specific transfer RNA; Sigma).

FAM-labeled DNA sequences were used for the KMnO_4_ oxidation of the thymine bases. KMnO_4_ at a final concentration of 1 mM was added to a DNA solution at 20°C, and the oxidation was stopped by adding an equal volume of a stop solution containing 1.5 M sodium acetate (pH 7.0), 1 M *β*-mercaptoethanol and 4 µg of tRNA. The DNA was then precipitated overnight with ethanol at −84°C. The precipitated DNA was dissolved in 1.5 M sodium acetate, mixed with 1 M piperidine and heated at 90°C for 1 h. After the ethanol precipitation, positions of the oxidized thymines in a FAM-labeled DNA sequence were determined by 20% PAGE containing 7 M urea using a TBE buffer [0.1 M Tris–HCl, 83 mM boric acid and 1 mM Na_2_EDTA, (pH 8.3)]. The fluorescent DNA on the gel was visualized using a fluorescence image analyzer (FLA-5100, Fujifilm). The rate constant (*k*) for oxidation was determined from a plot of the percentage cleaved (*f*) versus time (*t*), by fitting to a single exponential equation: *f* = *f*_max_[1 – exp(–*kt*)], where *f*_max_ is the percentage cleaved at infinite time. The modification experiments were performed in two or three independent trials, and the amounts of the cleaved fragments and the rate constant were determined from the average of these independent trials.

Cy3-labeled DNA sequences were used for the CMCT modification experiments that monitor the solvent accessibility of thymine and guanine bases (8). After incubation with 100 mM CMCT at 20°C for 24 h, a 5-fold volume of an acidic solution containing 0.3 M sodium acetate (pH 5.2) and 4 µg of tRNA was added. The DNA was precipitated overnight with ethanol at −84°C. The precipitated DNA was dissolved in 0.3 M sodium acetate and applied to PAGE to detect the DNA bands corresponding to an increase in the molecular weight by the coupling reaction with CMCT. In the cases of the CMCT modification for long DNA sequences, the primer extension reaction was performed, in which the CMCT-modified template strand interferes with the DNA primer extension catalyzed by DNA polymerase. In the experiment, after the reaction with CMCT at 20°C for 5 h, an excess amount of a non-labeled strand that facilitates the hybridization between the CMCT-modified DNA template and a fluorescently labeled primer was added. The primer extension was performed using 2.5 units of T7 DNA polymerase (Sequenase version 2.0, USB) and a deoxynucleoside triphosphate mix [deoxynucleotide triphosphate (dNTP) mix: deoxyadenosine triphosphate (dATP), deoxyguanosine triphosphate (dGTP), deoxycytidine triphosphate (dCTP) and deoxytymidine triphosphate (dTTP) at 120 µM each], followed by PAGE to analyze the length of the extended products.

The chemical modification in the presence of the antibiotic netropsin or distamycin (Sigma) was performed with KMnO_4_ for 3 min or CMCT for 24 h at 20°C after incubation with the antibiotics at 10 µM for 30 min. A synthetic polyamide, Abu-Py-Py-Py, was prepared by manual solid-phase synthesis, using 9-fluorenylmethoxycarbonyl-protected 4-aminobutyric acid (Abu; Wako) and 9-fluorenylmethoxycarbonyl-protected 1-methylpyrrole (Py; Wako). The synthesized compound was purified by HPLC. Chemical modifications in the presence of Abu-Py-Py-Py were performed and analyzed as described for those using the antibiotics.

### Thermal melting curve of DNA duplexes and thermodynamic analysis

Ultraviolet (UV) absorbance of DNA was measured by a spectrophotometer (UV-1700, Shimadzu) equipped with a temperature controller. The extinction coefficient of an oligonucleotide was calculated on the basis of the nearest-neighbor approximation, and those of the nucleotide derivatives were assumed to be the same as that of the corresponding natural nucleotides. A thermal melting curve in a buffer containing 1 M NaCl, 10 mM Na_2_HPO_4_ and 1 mM Na_2_EDTA, adjusted to pH 7.0, was monitored at 260 nm at the rate of 0.5 or 1°C min^−^^1^. The melting temperature (*T*_m_) at which half of a duplex structure was denatured was determined from the melting curve. The thermodynamic parameters Δ*H*°, Δ*S*° and the free energy change at 37°C (Δ*G*°) for DNA duplex formations were determined from the *T*_m_ obtained at different total strand concentrations (*C*_t_) using a plot of *T*_m_^−^^1^ versus log (*C*_t_/4) and from the melting curves fit to a theoretical equation assuming a two-state transition (6,9).

### DNA ligase reaction

A FAM-labeled 24mer DNA (a final concentration of 5 µM) and an 11mer DNA (10 µM) were heated with a 35mer DNA template strand (10 µM) to 90°C and then cooled to 20°C at the rate of −1°C min^−^^1^ before use. The ligation creating a phosphodiester bond between the 3′-hydroxyl group of the 24mer DNA and the 5′-phosphate group of the 11mer DNA was performed using 0.25 units of T4 DNA ligase (Promega) in a reaction buffer containing 30 mM Tris–HCl (pH 7.8), 10 mM MgCl_2_, 10 mM dithiothreitol (DTT) and 1 mM ATP at 20 °C. The reaction was terminated by adding at least a 4-fold volume of formamide loading solution containing 10 mM Na_2_EDTA. The mixed solution was then heated at 90°C for 3 min and loaded on 15% PAGE containing 7 M urea using the TBE buffer, followed by analysis using the fluorescence image analyzer. The reaction rate constant was determined by a non-linear regression of the time course data fit to a single exponential curve. The ligation rates determined from averages of two or three independent experiments were compared.

### DNA polymerase reactions

The Klenow fragment of *Escherichia coli* DNA polymerase I without 3′→5′ exonuclease activity (Klenow fragment *exo*^−^, Promega) and the T7 DNA polymerase that was genetically engineered to abolish its 3′→5′ exonuclease activity (Sequenase version 2.0) were used. The synthesis of 5′-*O* triphosphate of dA^phe^ (dA^phe^TP) is described in the Supplementary Data. The polymerase reaction was performed with a FAM-labeled primer (5 µM) and a template (10 µM). The DNA strands were heated to 90°C and then cooled to 37°C at the rate of −1°C min^−^^1^ before use. After adding 1 unit of the Klenow fragment or 5 units of the T7 DNA polymerase, the reaction of a single base extension was initiated by adding a complementary dNTP (either dATP, dGTP, dCTP or dTTP) at 120 µM to the reaction buffer containing 50 mM Tris–HCl (pH 7.2), 10 mM MgSO_4_ and 0.1 mM DTT, unless otherwise stated. The polymerase reaction was also performed with a dNTP mix (dATP, dGTP, dCTP and dTTP at 120 µM each) to extend a DNA primer to a full-length product. Quenching and analysis of the reaction rate were performed like those described for the ligase reaction. The experiments were performed in two or three independent trials, and the catalytic turnover number (*k*_cat_) and Michaelis constant (*K*_m_) were obtained from a Hanes–Woolf plot.

## RESULTS AND DISCUSSION

### Conformation of thymine bases opposite the 2′-deoxyadenosine lesions

The DNA adduct lesions illustrated in Figure 1A have either a phenyl or a naphthyl group but retain the hydrogen bonding sites for Watson–Crick base pairing. Our previous studies using the UV thermal melting curves and fluorescence spectra suggested that dA^phe^ and dA^naph^ introduced into a DNA sequence do not form a base pair with thymine in a complementary strand but induce base flipping (6,7). These spectroscopic measurements, however, only provide suggestive evidence for the base conformation. In this study, we examined the chemical modifications to directly assess the conformation of DNA bases. We used KMnO_4_ that oxidizes the 5,6-double bond of thymine, followed by treatment with piperidine to cleave a DNA strand at the oxidized thymine residues. We also used a soluble carbodiimide compound, CMCT, which forms a covalent bond with the *N*3 of thymine. The modification with CMCT increases the molecular weight of a DNA and shifts its mobility in the polyacrylamide gel (8). Because KMnO_4_ and CMCT preferentially react with thymines in an unstacked conformation, only the bases being perturbed in an extrahelical conformation are highly modified. Accordingly, these chemical modification experiments have benefits in terms of providing the positions and numbers of unstacked thymines in a DNA strand.

**Figure 2.**
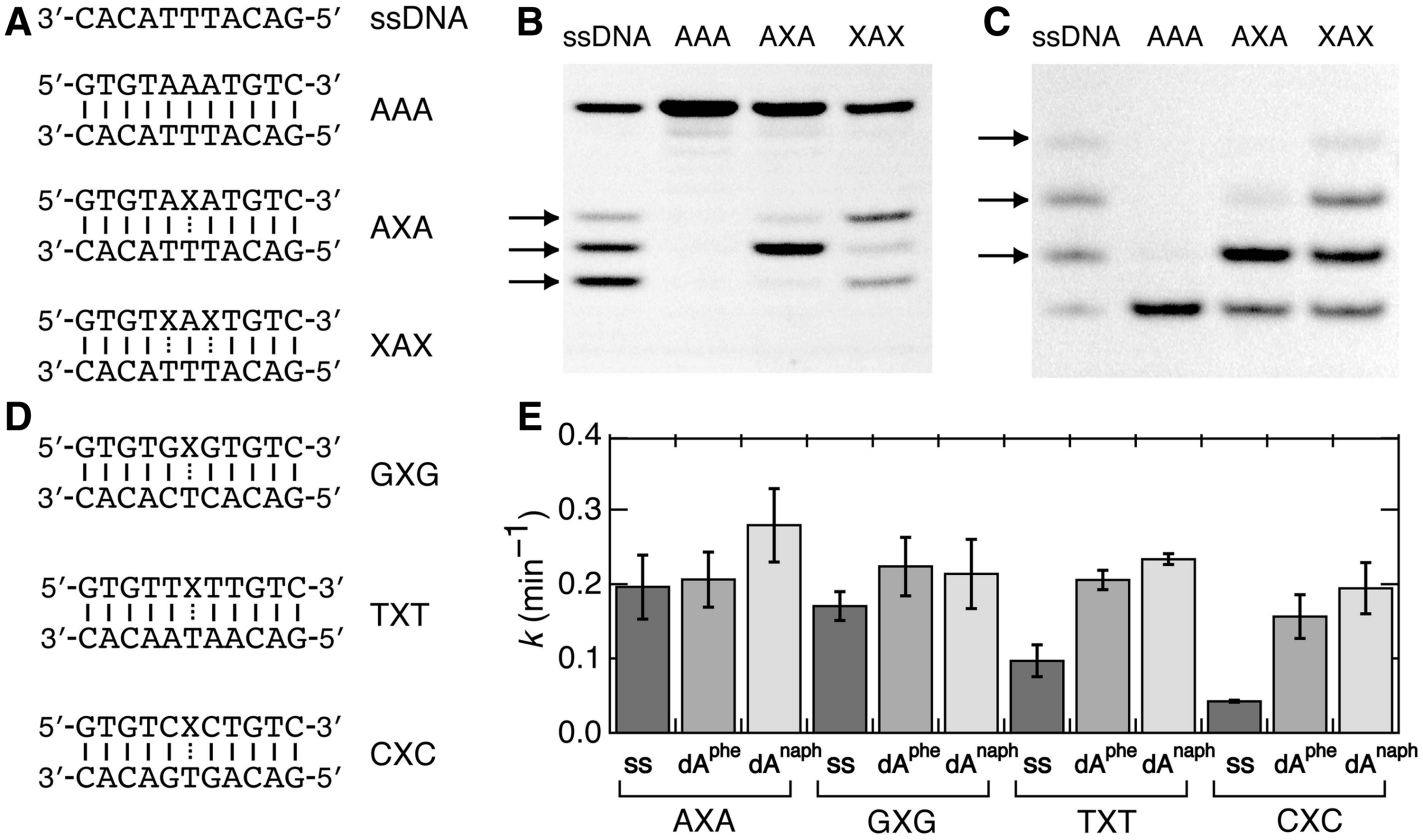
(**A**) DNA sequences used for the chemical modification experiments and their abbreviations. The bottom strands of the duplexes are labeled with FAM or Cy3. X represents the position where the nucleotide derivative is introduced. (**B**) PAGE for the KMnO_4_ oxidation of thymines opposite dA^phe^ introduced at the X position. The cleaved DNA fragments are indicated by arrows. (**C**) PAGE for the CMCT modification of thymines opposite dA^phe^. The modified DNA strands are indicated by arrows. (**D**) DNA sequences having different base pairs adjacent to the X/dT pair. (**E**) Rate constants for the KMnO_4_ oxidation of thymines opposite dA^phe^ or dA^naph^, compared with those for the corresponding single-stranded DNAs (ss). The value for the single-stranded DNA containing three thymines represents the average of the cleavage of a single thymine.

Figure 2A shows the 11mer oligonucleotides, forming dA/dT and X/dT pairs in the middle of the sequences, used for the chemical modifications. There were three thymines in the strand labeled with a fluorophore at the 5′-end. The PAGE analysis shown in Figure 2B provided that the single-stranded DNA yielded three cleaved fragments, corresponded to the cleavage at each thymine residue, after the treatments with KMnO_4_ and piperidine. The DNA duplexes containing one or two dA^phe^ residues at the X position yielded one or two cleaved fragments, respectively, whereas the fully matched natural duplex yielded no cleaved fragment. These results indicate unstacking of the thymine bases located opposite dA^phe^. In contrast, the dT/dT, dC/dT and dG/dT mismatch pairs produced no cleaved fragment (Figure S1), suggesting at least a partially stacked conformation of the mismatched thymine bases. The PAGE shown in Figure 2C provides the numbers of thymines modified with CMCT, and the results are consistent with a structural perturbation of the thymine bases opposite dA^phe^. It is thus probable that the phenyl group of dA^phe^ intercalates into the duplexes and induces unstacking of a thymine base without substantially perturbing adjacent dA/dT base pairs.

The oxidation rates were measured to study the dynamics of thymine bases. Because adjacent base pairs affect the strength of the stacking interaction, DNA duplexes with different base pairs next to an X/dT pair (Figure 2D) were also compared. For the single-stranded DNAs, thymines adjacent to the purine nucleotides showed slower modification rates than those adjacent to the pyrimidine nucleotides (Supplementary Figure S2) because of stronger stacking interactions with the purine bases. Remarkably, regardless of the type of adjacent base pairs, the oxidation rates of the thymines opposite dA^phe^ and dA^naph^ were similar to each other and comparable with or higher than those of the single strands (Figure 2E). The observation suggests an unconstrained conformation of these thymine bases. We concluded that the 2′-deoxyadenosine lesions do not form a base pair with thymine in a DNA duplex, although there may be an equilibrium with the base pair conformation as minor components.

### Conformation of guanine bases opposite the 2′-deoxycytidine lesions

Previous thermodynamic studies have proposed that guanine forms a base pair with dC^phe^ in a complementary strand (10). In this study, we first compared the thermodynamic parameters for the formations of the DNA duplexes, 5′-GTGTCXCTGTC-3′/5′-GACAGNGACAC-3′ (N = dA, dG, dC, dT, or the tetrahydrofuran abasic analog dF) containing dC, dC^phe^ or dC^naph^ at the X position. The duplex stability increased when dC^naph^ was introduced opposite dA, dC, dT or dF (Supplementary Table S1), as observed in the case of dC^phe^. The change from dC^phe^ to dC^naph^ stabilized the duplex by 1–2 kcal mol^−^^1^ in Δ*G*°. These thermodynamic data are consistent with unstacking of the adenine, cytosine and thymine bases owing to the naphthyl group stacking of dC^naph^.

**Figure 3.**
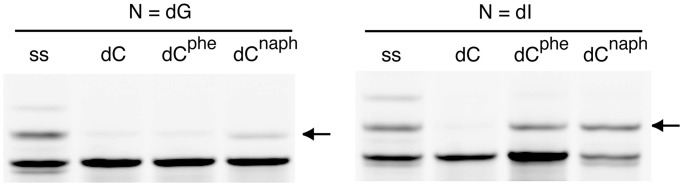
CMCT modifications for dG (left) or dI (right) in the duplex 5′-GTGTCXCTGTC-3′/5′-GACAGNGACAC-3′ (X = dC, dC^phe^ or dC^naph^) or the single-stranded 5′-GACAGNGACAC-3′ (ss). Arrows indicate the DNA bands corresponding to an increase in the molecular weight of the Cy3-labeled sequence containing N (dG or dI) owing to the reaction with CMCT.

In contrast, introducing dC^naph^ in place of dC located opposite the guanine decreased the duplex stability by 1.0 kcal mol^−^^1^. The chemical modification using CMCT, which reacts with the *N*1 of guanine, showed no modification of the guanine opposite dC or dC^phe^, but only a slight reaction product with dC^naph^ (Figure 3, left). We also examined the DNA sequences containing 2′-deoxyinosine, dI. The inosine base lacks the 2-amino group of guanine, and it can form a base pair with dC through two hydrogen bonds. However, the inosine bases of the dC^phe^/dI and dC^naph^/dI pairs, but not that of dC/dI, were effectively modified with CMCT (Figure 3, right). The data indicate a considerable presence of unstacked conformations of the inosine bases opposite the 2′-deoxycytidine lesions. It is possible that dC^phe^ and dC^naph^ change their conformations in accordance with the difference in the interaction energies between the stacking of the attached aromatic group and the base pairing through hydrogen bonds.

### Conformation of dA^phe^ during enzyme reactions

As the DNAs used for the ligase and polymerase reactions have relatively long sequences, their base conformations were investigated using the primer extension assay. According to the results using CMCT by which the modified sites prevent the DNA primer extension by DNA polymerase, it was concluded that dA^phe^ and dA^naph^ induced thymine unstacking, dC^phe^ formed a base pair with guanine, dC^naph^ partially induced guanine unstacking and dC^phe^ and dC^naph^ induced inosine base unstacking (Supplementary Figure S3). The observations agreed with the data obtained with the short sequences described earlier in the text.

**Figure 4.**
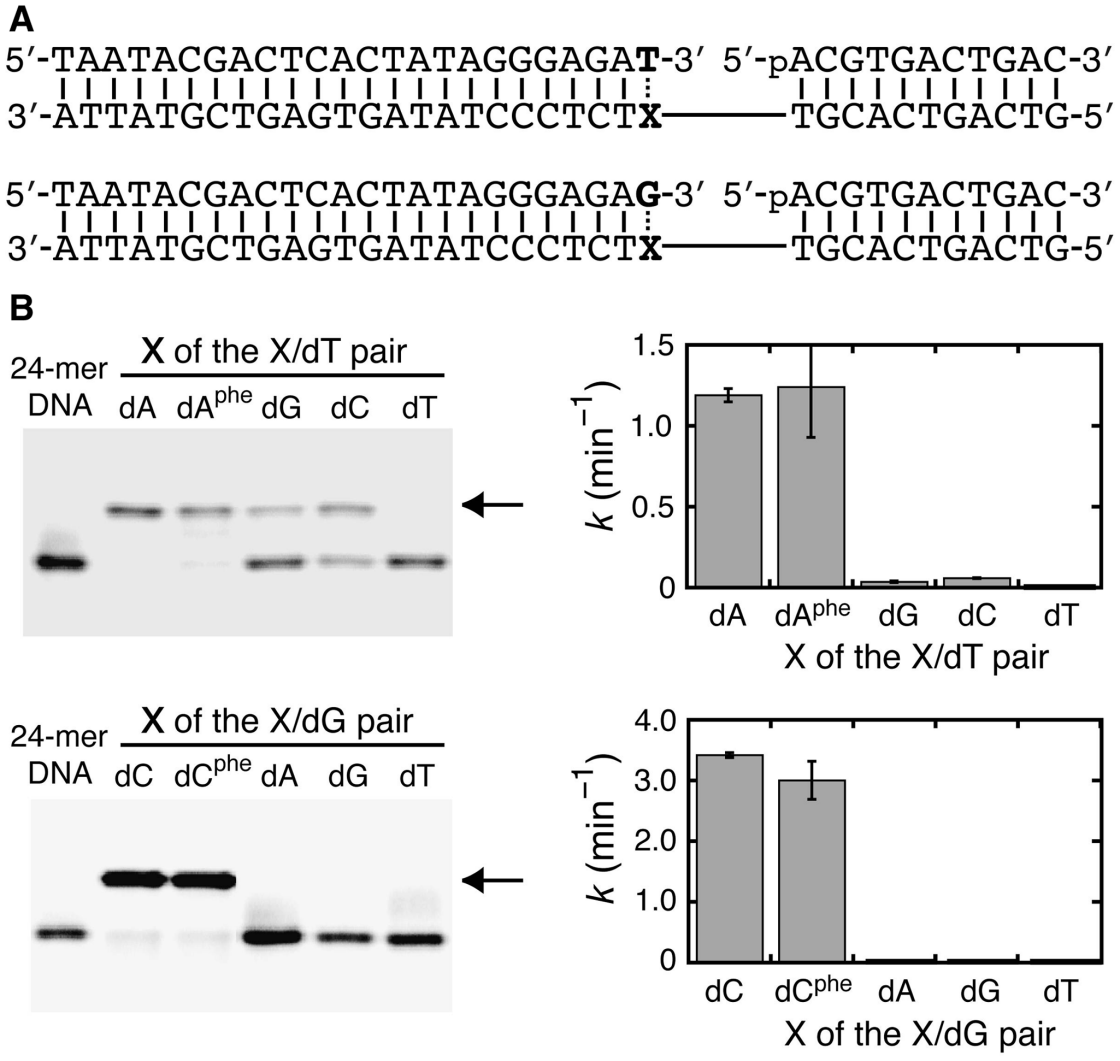
(**A**) DNA sequences forming an X/dT or X/dG pair at the 3′-end of the junction used for the T4 DNA ligase reaction. The 24mer strand to be ligated was labeled with Cy3 at the 5′-end, and the 11mer strand had a phosphate group (denoted by p) at the 5′-end. (**B**) PAGE representing the fluorescent DNAs after the ligation reaction for 5 min (arrows indicate the ligated DNA strand), and the ligation rate constants for the DNA sequences forming an X/dT or X/dG pair.

T4 DNA ligase catalyzes the covalent bond formation between the 5′-phosphate and 3′-hydroxyl group ends of different DNA strands. Previous biochemical studies have suggested that the hydrogen bond acceptors in the DNA minor groove are crucial for the ligation, and the reaction efficiency is significantly reduced when the terminal base pair on the 3′-end of the ligation junction is disrupted (11,12). We used T4 DNA ligase and analyzed the ligation efficiency of DNA fragments hybridized with the DNA template containing dA^phe^ or dC^phe^ at the 3′-end of the junction (Figure 4A). We found that the ligation rates for the DNA strands forming a terminal dA^phe^/dT or dC^phe^/dG pair were fast and similar to those forming a Watson–Crick dA/dT or dC/dG base pair, respectively, whereas the pairs of dG/dT, dC/dT, dT/dT, dA/dG, dG/dG and dT/dG produced low ligation activities (Figure 4B). The high ligation efficiency with the DNA adduct lesions suggests the formation of base pairing between dA^phe^ and thymine as well as between dC^phe^ and guanine during the ligation reaction.

**Figure 5.**
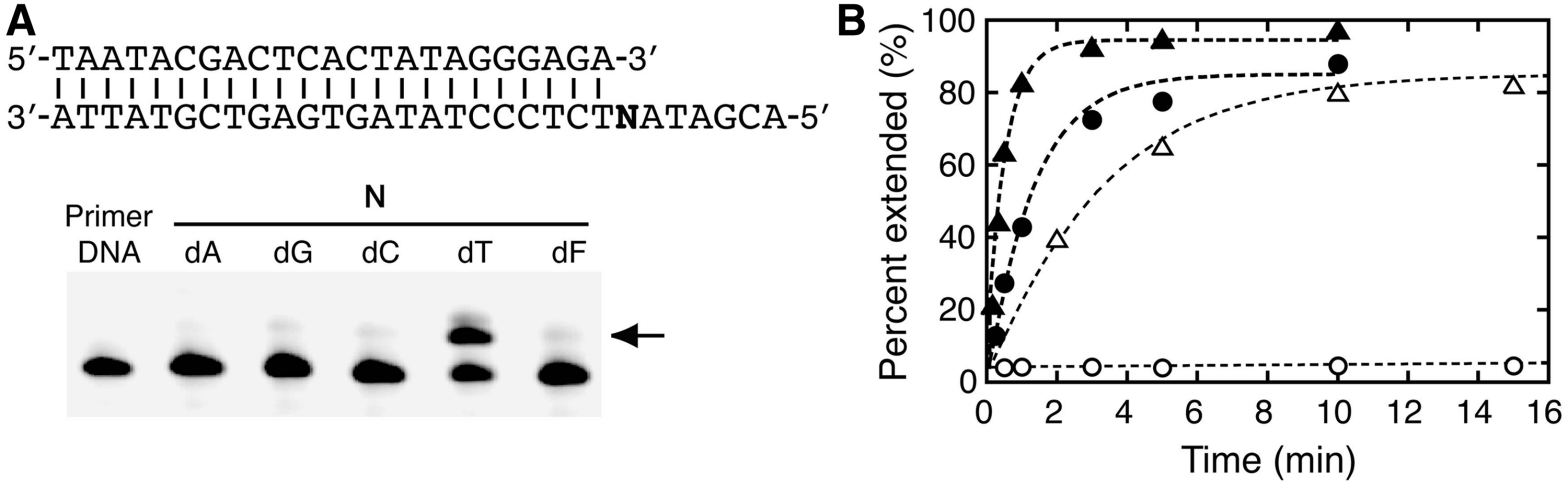
(**A**) DNA sequences and PAGE for the incorporation of dA^phe^TP opposite N (dA, dG, dC, dT or dF) in the DNA template by the Klenow fragment (0.45 units). The reaction was conducted with 500 µM dA^phe^TP for 2 min. The arrow indicates the extended DNA primer. (**B**) Kinetic traces for the primer extension incorporating dA^phe^TP (circles) and dATP (triangles) opposite dT (closed symbols) or dF (open symbols) by the Klenow fragment.

We also investigated the polymerase reactions. It is known that the shapes and sizes are important for the polymerization with non-natural nucleotides (13). We prepared the 5′-*O* triphosphate of dA^phe^ (dA^phe^TP) by oxidative treatment of its *H*-phosphonate monoester through silyl phosphite (14–16) (Supplementary Figure S4) and incorporated it into a DNA primer using the Klenow fragment of *E. coli* DNA polymerase I without 3′→5′ exonuclease activity. This polymerase strictly recognizes a dNTP according to the rules of Watson–Crick base pairing or selects a large hydrophobic base analog opposite an abasic site in the DNA template (17–20). Incorporation of dA^phe^TP opposite an abasic site is expected if the phenyl group of dA^phe^ preferentially adopts the stacking conformation. However, the Klenow fragment incorporated dA^phe^TP opposite thymine, but not opposite the abasic site (Figure 5A). The rate constant for the dA^phe^TP incorporation opposite thymine was lower, but significant, than that for the reaction with dATP (0.77 and 1.9 min^−^^1^, respectively). The result suggests that the incoming dA^phe^TP binds to the polymerase and forms the base pair with thymine. As shown in Figure 5B, the incorporation of dA^phe^TP opposite the abasic site was markedly inhibited (<0.001 min^−^^1^), whereas the incorporation of dATP was substantial (0.32 min^−^^1^); referred to as the A-rule (21,22).

The enzyme kinetic parameters summarized in Table 1 show that the Michaelis constant, *K*_m_, for the incorporation opposite thymine increases >20-fold when dATP is changed to dA^phe^TP, but their values of the catalytic turnover number, *k*_cat_, differ by only 2-fold. The second-order rate constant, *k*_cat_/*K*_m_, for the incorporation of dA^phe^TP opposite thymine is 2000-fold higher than that for the incorporation opposite the abasic site, and the number is much higher than that for the reaction using dATP (46-fold). It is emphasized that these results are inconsistent with the chemical modification data showing an unstacked conformation of the thymines opposite dA^phe^.

**Table 1.** Kinetic parameters for the incorporation of a dNTP by the Klenow fragment

dNTP	N in the DNA template	*k*_cat_ (min^−1^)	*K*_m_ (µM)	*k*_cat_/*K*_m_ (min^−1 ^M^−1^)
dATP	dT	1.9 ± 0.1	11 ± 4	(1.7 ± 0.6) × 10^5^
dA^phe^TP	dT	0.94 ± 0.01	230 ± 20	(4.2 ± 1.0) × 10^3^
dATP	dF	0.41 ± 0.02	110 ± 20	(3.7 ± 0.8) × 10^3^
dA^phe^TP	dF	0.044 ± 0.007	(2.1 ± 0.3) × 10^4^	2.1 ± 0.5

### DNA polymerase reactions downstream of the lesion site

**Figure 6.**
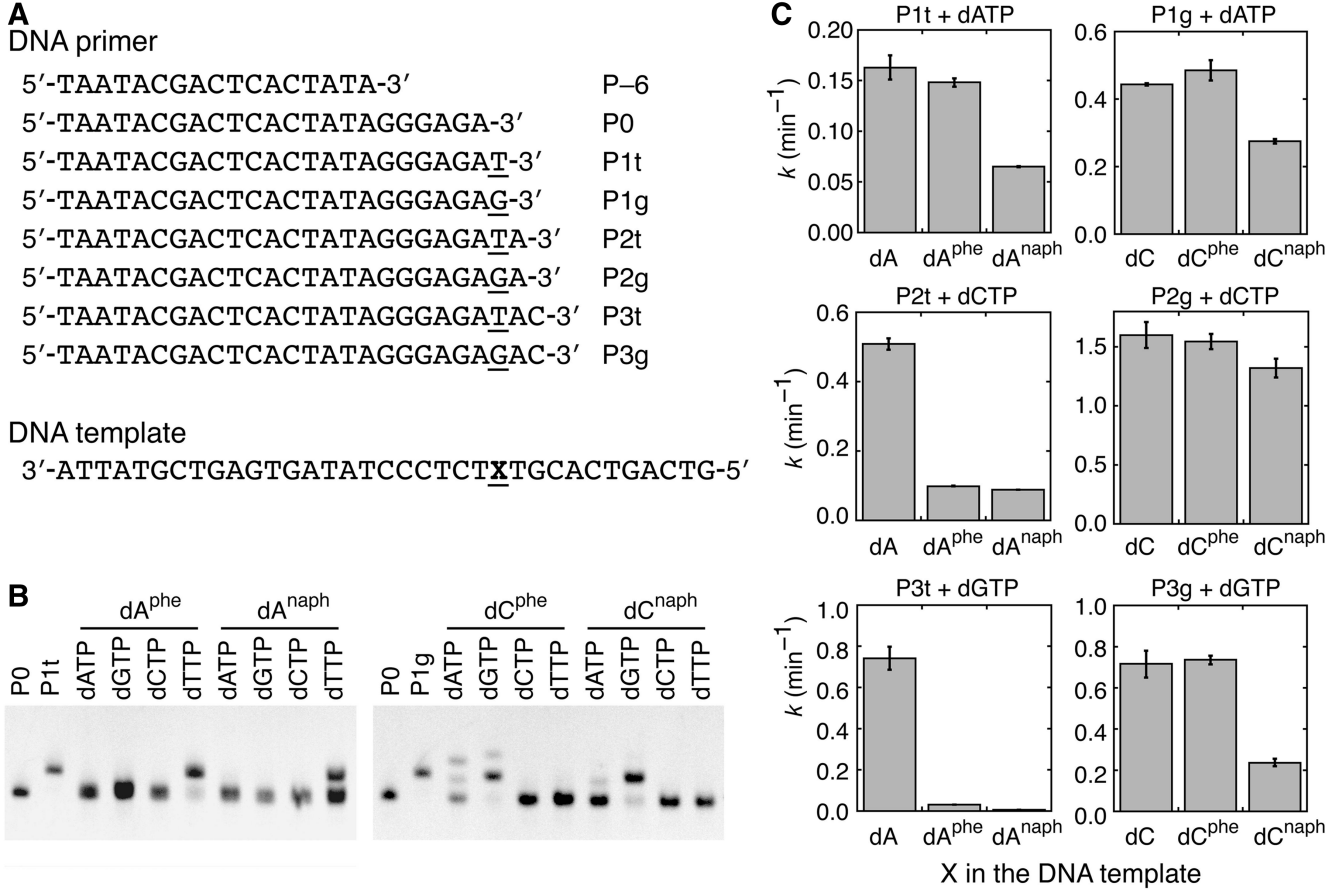
(**A**) DNA sequences used to measure the activity of the DNA polymerases. (**B**) PAGE for the incorporation of dNTP opposite the 2′-deoxyadenosine and 2′-deoxycytidine derivatives by the Klenow fragment. The reaction was conducted with P0 and 120 µM dNTP for 10 min. (**C**) Rate constants of the primer extensions by the Klenow fragment with 120 µM dNTP (dATP, dCTP or dGTP) using different primers and DNA templates.

Influences of the adduct lesions introduced into a template strand on the polymerase reactions were evaluated using different primer lengths, 17mer and 23–26mer DNAs, presented in Figure 6A. The polymerization with P–6 and a dNTP mix (dATP, dGTP, dCTP and dTTP) using the Klenow fragment was run through the lesion site on the template strand: the reaction gave the full-length products, except for the DNA template including dA^naph^ that effectively stopped the reaction 3 nt downstream of the lesion site (Supplementary Figure S5A). Inhibition of the downstream replication was previously reported for a mutagenic adduct of benzo[a]pyrene (23).

To investigate the influence on each incorporation step, the single base extensions by the Klenow fragment were measured. It was found that the reaction using primer P0 was selective for dTTP opposite dA^phe^ and dA^naph^ or for dGTP opposite dC^phe^ and dC^naph^ (Figure 6B). The same selectivity was also observed when T7 DNA polymerase was used (Figure S5B). The kinetic parameters shown in Table 2 indicate that the adduct lesions in the DNA template change the *K*_m_ rather than the *k*_cat_, as observed for the incorporation of dA^phe^TP.

**Table 2.** Kinetic parameters for the incorporation of a complementary dTTP or dGTP using primer P0 and the DNA template containing X^a^

	Klenow fragment			T7 DNA polymerase		
X in the DNA template	*k*_cat_ (min^−1^)	*K*_m_ (µM)	*k*_cat_/*K*_m_ (min^−1^M^−1^)	*k*_cat_ (min^−1^)	*K*_m_ (µM)	*k*_cat_/*K*_m_ (min^−1^M^−1^)
dA	0.36 ± 0.01	3.8 ± 0.9	(9.5 ± 2.3) × 10^4^	3.5 ± 0.1	3.3 ± 0.5	(1.1 ± 0.2) × 10^6^
dA^phe^	0.30 ± 0.01	19 ± 3	(1.6 ± 0.2) × 10^4^	2.1 ± 0.1	39 ± 5	(5.4 ± 0.8) × 10^4^
dA^naph^	0.27 ± 0.01	94 ± 8	(2.8 ± 0.3) × 10^3^	1.3 ± 0.2	(1.4 ± 0.2) × 10^3^	(9.1 ± 1.7) × 10^2^
dC	1.6 ± 0.1	8.6 ± 2.3	(1.9 ± 0.5) × 10^5^	4.3 ± 0.1	3.2 ± 0.4	(1.3 ± 0.2) × 10^6^
dC^phe^	1.4 ± 0.1	14 ± 3	(1.0 ± 0.2) × 10^5^	2.3 ± 0.1	59 ± 8	(3.9 ± 0.5) × 10^4^
dC^naph^	0.98 ± 0.07	69 ± 12	(1.4 ± 0.3) × 10^4^	1.9 ± 0.2	(4.2 ± 0.4) × 10^3^	(4.4 ± 0.6) × 10^2^

^a^DNA templates containing dA, dA^phe^ or dA^naph^ at the X position were examined for the incorporation of dTTP, and those containing dC, dC^phe^ or dC^naph^ were examined for the incorporation of dGTP. The DNA sequences are given in Figure 6A.

Figure 6C compares the rate constants for the downstream reactions. The incorporation of dATP next to the X position was measured using primers P1t or P1g, which formed the X/dT pair or X/dG pair, respectively, at the end. Although the primer extensions with P1t next to the non-Watson–Crick dG/dT, dC/dT or dT/dT were slow (*k* = 0.0087–0.015 min^−^^1^; Supplementary Figure S5C), the reaction next to dA^phe^/dT (0.15 min^−^^1^) was as fast as the reaction next to the dA/dT base pair. The reaction next to the dA^naph^/dT pair was slower but still significant (0.065 min^−^^1^). The reactions with P1g next to dC^phe^/dG and dC^naph^/dG provided 0.49 and 0.28 min^−^^1^, respectively, comparable with that with dC/dG, but the pairs of dA/dG, dG/dG and dT/dG yielded values <0.03 min^−^^1^. These data strongly suggest the base pair formation between the lesion sites and their complementary bases next to the dNTP incorporation site.

When the longer primers were used, several non-Watson–Crick base pairs, e.g. dC/dT and dT/dG formed at the X position did not significantly inhibit the primer extension (Supplementary Figure S5C). The primer extension starting 2 nt downstream of dC^phe^ or dC^naph^ were comparable with that for the reaction downstream of dC, whereas dA^phe^ and dA^naph^ significantly decreased the rate. For the primer extension starting 3 nt downstream of the X position, dC^phe^ did not affect the rate, but other adduct lesions decreased the rate. Overall, the rate reductions were more evident with the 2′-deoxyadenosine lesions and the naphthyl group adducts. In particular, dA^naph^ yielded less reaction product with primer P3t, consistent with the results of the primer extension using P–6 and the dNTP mix.

According to the reported high-resolution crystal structure and mutation studies, the polymerases interact with the DNA minor groove through the *N*3 of purine bases and the *O*2 of pyrimidine bases forming a Watson–Crick base pair (24–26). In contrast, there is no obvious interaction, and there is sufficient space in the major groove for accommodating the phenyl and naphthyl groups in an extrahelical conformation. It is probable that the polymerase reaction is inhibited when the *O*2 of a complementary base becomes unavailable for the minor groove interaction by the intercalation of the bulky groups into the duplex. The crystal structure shows the presence of minor groove interactions within the 5 bp from the dNTP incorporation site (24–26), but we observed less primer extension 3 nt downstream of dA^naph^. This result may reflect a possibility that the minor groove interactions away from the protein interior are relatively weak and overcome by the naphthyl group stacking.

### DNA replication with modified residues

Hydrophobic base analogs that have the ability to replicate and undergo evolution have promise for expanding their biotechnological and clinical potentials, such as the polymerase chain reaction and systematic evolution of ligands by exponential enrichment. It is known that some non-natural deoxynucleoside triphosphates, which do not form hydrogen-bonded base pairs, can be used as substrates for DNA polymerases, including the Klenow fragment, T4 DNA polymerase, DNA polymerase *α* and a genetically manipulated chimeric polymerase, if their shapes and sizes conform to a partner base or the abasic site in a template strand (17–21,27–32). A rigid conformation brought about by introducing a planar aromatic ring system is preferred for the purposes of shape complementarity and high stacking energy because of reductions in the conformational space and entropy loss. In contrast, the aromatic adduct lesions having rotatable bonds presented in Figure 1A can change their conformations to be compatible for the polymerase reactions. The result is consistent with the previous reports that DNA lesions produced by exposure to isocyanate compounds appear to have little or no ability to induce gene mutations in a cell (2–4). It is possible that a rotation of the ureido linker allows the conformational change that enables gene replication without significant mutations. Some other bulky DNA adducts having rotatable bonds may also have some capability of DNA replication.

### Conformational changes induced by minor groove-binding molecules

The enzyme experiments suggested that the minor groove interactions stabilize the base pair conformation between the 2′-deoxyadenosine lesions and thymine. To verify this model, minor groove-binding netropsin and distamycin were examined. The compounds selectively bind to consecutive dA/dT base pair sites through the hydrogen bonds with the *N*3 of adenine and with the *O*2 of thymine (33–37); these interaction sites are the same as those of the DNA polymerases.

**Figure 7.**
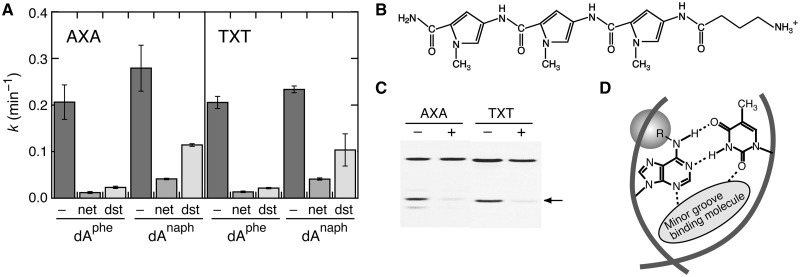
(**A**) Rate constants for the KMnO_4_ oxidation of thymines opposite dA^phe^ or dA^naph^ in AXA or TXT in the absence (−) and presence of netropsin (net) or distamycin (dst). (**B**) Chemical structure of a synthetic polyamide, Abu-Py-Py-Py. (**C**) PAGE for the KMnO_4_ oxidation of thymines opposite dA^phe^ in AXA or TXT in the absence (−) or presence (+) of Abu-Py-Py-Py. (**D**) A pictorial representation of the base pairing between the 2′-deoxyadenosine lesions (R represents a phenylcarbamoyl or naphthylcarbamoyl group) and thymine in a DNA duplex, stabilized by interactions with a minor grove binding molecule.

The DNA duplexes, AXA and TXT, offer the binding sites for netropsin and distamycin if the 2′-deoxyadenosine lesions form the base pair with thymine. The binding to the duplexes was confirmed by the DNase I footprinting experiments, and the melting temperature increased by ∼10°C after the addition of netropsin or distamycin at 10 µM. We found that these compounds reduced the efficiency of the KMnO_4_ oxidation of thymines opposite dA^phe^ and dA^naph^ in AXA and TXT, whereas the reduction was not observed with GXG and CXC that did not have consecutive dA/dT base pairs (Supplementary Figure S6). The experiments using CMCT also gave the same results (data not shown). Figure 7A shows that netropsin more effectively reduced the oxidation efficiency than distamycin, consistent with its higher binding capacity to a target DNA (37). Moreover, a synthetic polyamide, Abu-Py-Py-Py, comprising three pyrrole rings with a positive charge presented in Figure 7B, which was designed to bind to consecutive dA/dT base pair sites (38,39), also reduced the oxidation efficiency of AXA and TXT (Figure 7C). It can be concluded that the conformation of thymine opposite the 2′-deoxyadenosine lesions changes owing to the interactions with the minor groove-binding molecules (Figure 7D).

## CONCLUSION

The DNA adduct lesions produced by the reaction with aromatic isocyanates are accommodated into a DNA duplex with a minimum disruption of the double helical conformation and the thermal stability, and they do not disrupt the DNA ligase and DNA polymerase reactions. The chemical modification and enzyme experiments showed that the adduct lesions have a capability of adopting dual conformations by changing the orientation of the attached aromatic group. It is also presented that the attached aromatic groups change their orientation by interacting with netropsin, distamycin and synthetic polyamide, which may be supported by future studies using computer modeling. The properties of forming two different conformations and changing the conformation on binding with minor groove binding molecules are characteristic of the adduct lesions having rotatable bonds. In agreement with the proposition that the conformation is determined by the difference in the interaction energies between stacking of the attached aromatic group and base pairing through hydrogen bonds, dC^phe^ preferentially formed a base pair with guanine while dA^naph^ had the highest propensity for adopting the stacking conformation. The results suggest the molecular basis of DNA replication across the bulky adduct lesions, although there may exist a DNA repair mechanism for these lesions in a cell. In addition to the significance for biological processes, these nucleotide derivatives may be used for enhancing the phenotypic diversity of DNA molecules (40). The molecular design using rotatable bonds and modifications of the attached aromatic group, such as by providing the property of fluorescence, photoactivation, cross-linking or strong stacking effects, would expand their applications for molecular biology studies and for developing nucleic acid drugs.

## SUPPLEMENTARY DATA

Supplementary Data are available at NAR Online, including [41,42].

## FUNDING

MEXT-Supported Program for the Strategic Research Foundation at Private Universities, 2009–2014 [S0901047]; the ‘Academic Frontier’ Project (2004–2009) [04F012]; the Hirao Taro Foundation of the Konan University Association for Academic Research. Funding for open access charge: MEXT-Supported Program for the Strategic Research Foundation at Private Universities.

*Conflict of interest statement.* None declared.

## Supplementary Material

Supplementary Data
