# Supplementary material for: Conformational changes of the phenyl and naphthyl isocyanate-DNA adducts during DNA replication and by minor groove binding molecules
Source: Nucleic Acids Res. 2013 Jul 19;41(18):8581–90. doi: 10.1093/nar/gkt608 (PMC3794578; doi:10.1093/nar/gkt608)
Supplement: Supplementary Data [file supp_41_18_8581__index.html]

Conformational changes of the phenyl and naphthyl isocyanate-DNA adducts during DNA replication and by minor groove binding molecules — Conformational changes of the phenyl and naphthyl isocyanate-DNA adducts during DNA replication and by minor groove binding molecules — Supplementary Data 

# Conformational changes of the phenyl and naphthyl isocyanate-DNA adducts during DNA replication and by minor groove binding molecules

## 

files

**Files in this Data Supplement:**

- Supplementary Data - pdf file
