# Supplementary material for: Conformational changes of the phenyl and naphthyl isocyanate-DNA adducts during DNA replication and by minor groove binding molecules
Source: Nucleic Acids Res. 2013 Jul 19;41(18):8581–90. doi: 10.1093/nar/gkt608 (PMC3794578; doi:10.1093/nar/gkt608)
Supplement: Supplementary Data [file supp_gkt608_nar-01070-d-2013-File002.pdf]

Conformational changes of the phenyl and naphthyl isocyanate-DNA adducts during DNA replication and by minor groove binding molecules

Shu-ichi Nakano, Yuuki Uotani, Yuichi Sato, Hirohito Oka, Masayuki Fujii, and Naoki Sugimoto

Faculty of Frontiers of Innovative Research in Science and Technology (FIRST), Frontier Institute for Biomolecular Engineering Research (FIBER), and Department of Chemistry, Faculty of Science and Engineering, Konan University, and Molecular Engineering Institute (MEI) and Department of Environmental and Biological Chemistry, Kinki University

SUPPLEMENTARY DATA

Table S1. Thermodynamic parameters of the 11-mer DNA duplexes, 5′-GTGTCXCTGTC-3′/5′-GACAGNGACAC-3′, in the 1 M NaCl-phosphate buffer[a]

X/N pair	$\Delta H°$ (kcal mol^{-1})	$\Delta S°$ (cal $mol^{-1}K^{-1}$)	$\Delta G°$ (kcal mol^{-1})	T_m (°C)
dC/dF	–64.3±3.9	–180±12	–7.8±0.2	45.2
dC/dA	–66.8±5.2	–189± 2	–8.1±0.1	49.0
dC/dG	–85.7±3.5	–235±10	–12.9±0.3	65.3
dC/dC	–61.6±3.8	–174±12	–7.6±0.1	47.5
dC/dT	–70.4±3.2	–200±10	–8.4±0.1	49.6
dC^{phe}/dF	–67.5±5.9	–185±18	–10.2±0.2	59.0
dC^{phe}/dA	–63.9±3.8	–176±12	–9.4±0.3	50.1
dC^{phe}/dG	–79.4±6.2	–218±19	–12.0±0.4	63.2
dC^{phe}/dC	–71.2±2.0	–196± 6	–10.4±0.2	59.0
dC^{phe}/dT	–68.2±5.8	–189±18	–9.8±0.2	55.8
dC^{naph}/dF	–75.1±4.8	–207±15	–11.4±0.2	60.8
dC^{naph}/dA	–70.7±4.0	–195±12	–10.5±0.2	58.9
dC^{naph}/dG	–80.7±5.3	–222±16	–11.9±0.3	62.7
dC^{naph}/dC	–79.4±4.0	–219±13	–11.6±0.2	61.4
dC^{naph}/dT	–80.7±5.3	–222±16	–11.9±0.3	62.7

[a] All experiments were carried out in the buffer containing 1 M NaCl, 10 mM Na_2HPO_4, and 1 mM Na_2EDTA at pH 7.0. Values are the average of the data from the T_m^{-1} vs. log (C_t/4) plot and the curve fitting. $\Delta G°$ represents the free energy change at 37 °C for the duplex formations. T_m was calculated at the total DNA strand concentration of 100 µM. The data for the pairs by dC or dC^{phe} are derived from the reference 10.

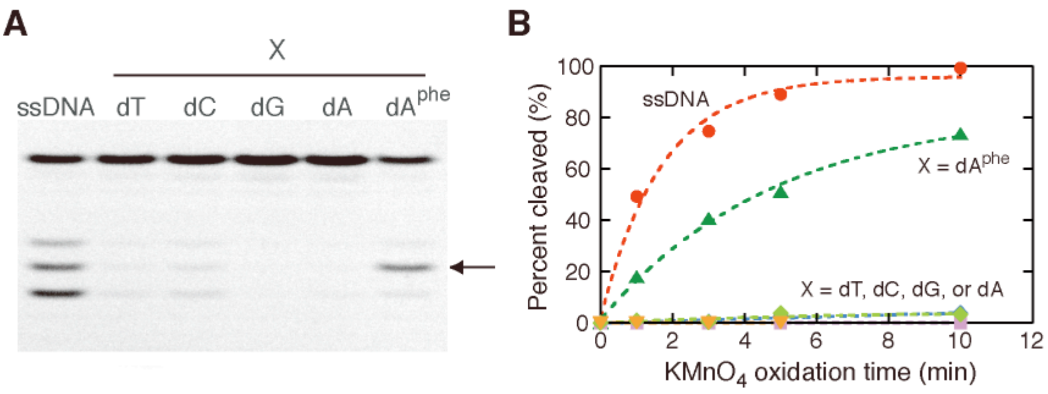

Figure S1. (A) PAGE for a single-stranded 5′-GACATTTACAC-3′ (ssDNA) and the duplexes 5′-GTGTAXATGTC-3′/5′-GACATTTACAC-3′ (X is dT, dC, dG, dA, or dA^{phe}) after the $KMnO_4$ oxidation for 1 min. (B) Kinetics for the oxidation of thymines in the ssDNA or the duplexes forming an X/dT pair.

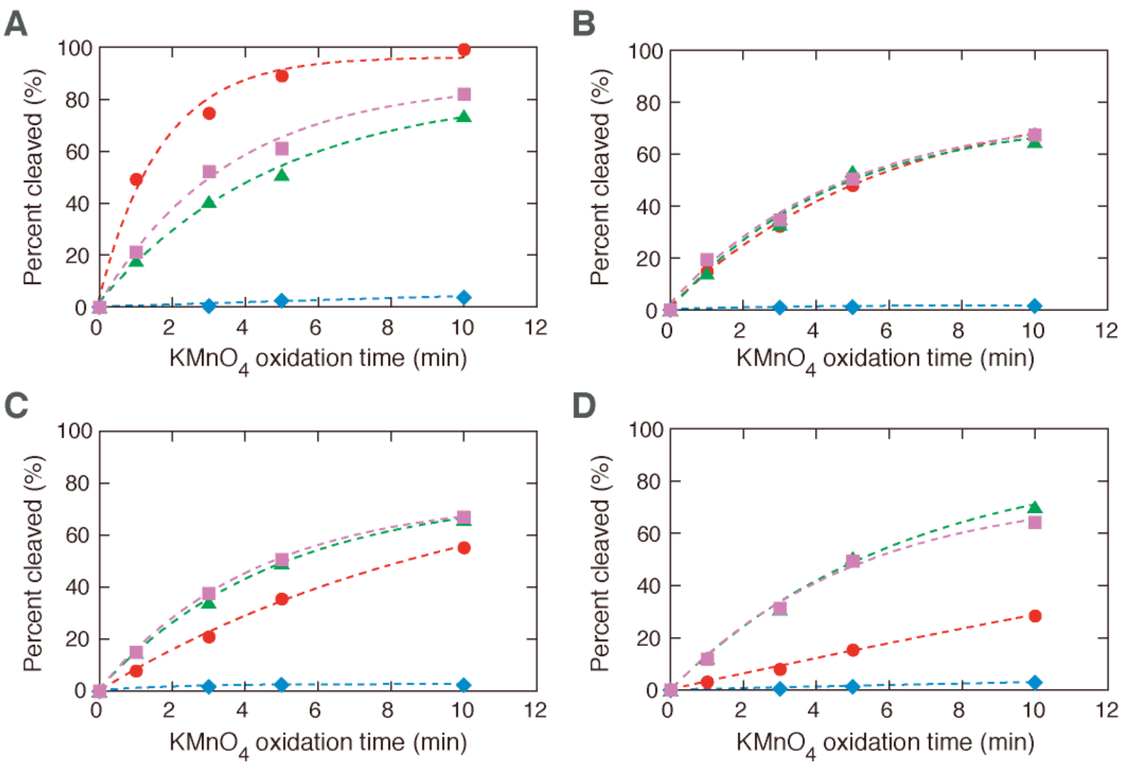

Figure S2. Kinetics for the $KMnO_4$ oxidation of thymines opposite X in the DNA duplexes, (A) 5′-GTGTAXATGTC/5′-GACATTTACAC-3′ (AXA), (B) 5′-GTGTGXGTGTC/5′-GACACTCACAC-3′ (GXG), (C) 5′-GTGTTXTTGTC/5′-GACAATAACAC-3′ (TXT), and (D) 5′-GTGTCXCTGTC/5′-GACAGTGACAC-3′ (CXC), where X is dA (blue), dA^{phe} (green), or dA^{naph} (purple). The data for single-stranded DNAs are in red. The kinetic trace of the single-stranded 5′-GACATTTACAC-3′ indicates the total amount of DNA fragments cleaved at either thymine residue.

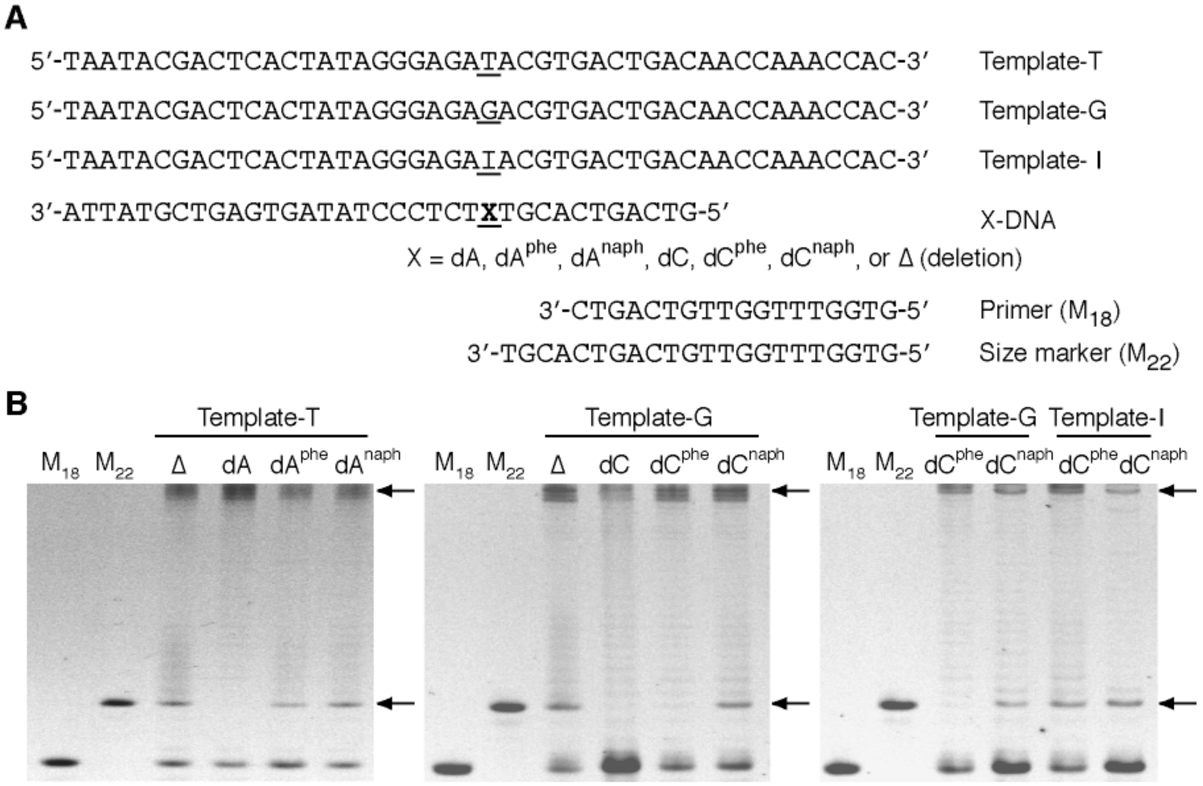

Figure S3. A. DNA sequences used for the primer extension experiments. CMCT was treated with the DNA template (Template-T, -G, or -I) hybridized with a complementary strand (X-DNA). The single nucleotide deletion at the X position (denoted by Δ) creates an unpaired bulge base of dT, dG, or dI. B. PAGE for the primer extension by T7 DNA polymerase using the CMCT-treated DNA templates and a FAM-labeled primer (M_{18}). The arrows indicate the fully extended product and the 22-mer products stopped at position dT, dG, or dI (underlined bases in panel A) in the template strands.

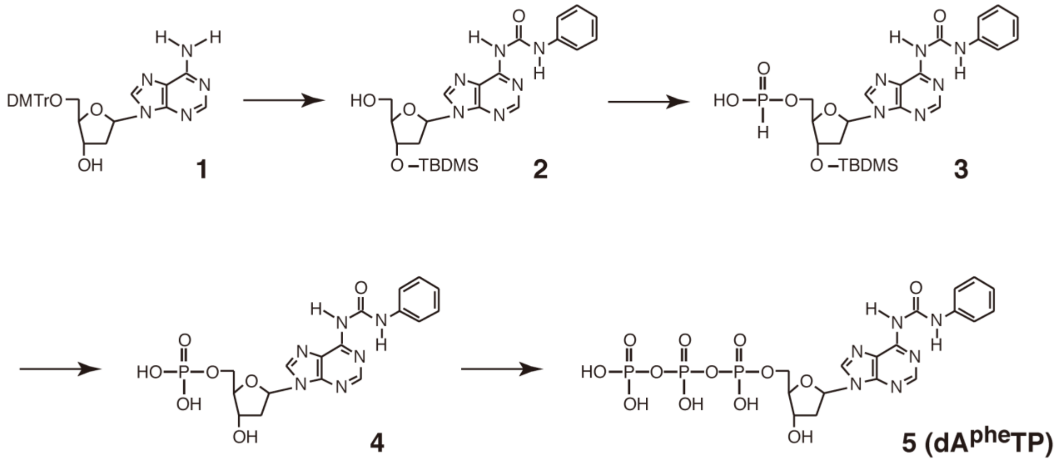

Figure S4. Synthesis of dA^{phe}TP. 5′-*O*-dimethoxytrityl (5′-*O*-DMTr)-2′-deoxyadenosine **1** in dry pyridine was silylated with *t*-butyldimethylsilyl chloride (TBDMSCl). After stirring at room temperature for 6 h, the 5′- and 3′-*O*-protected 2′-deoxyadenosine was reacted with *N*-phenylurea in CH_3CN under reflux for 6 h. After evaporation, the fully protected derivative was treated with 80% acetic acid for 1 h. The 5′-*O* free 2′-deoxyadenosine derivative **2** was obtained at an overall yield of 73% from **1**. Product **2** was converted into the 5′-*O* *H*-phosphonate derivative **3** (quantitative yield from **2**) as a colorless foam based on procedures reported previously (16). After deprotecting the 3′-*O* TBDMS group of **3** by treatment with tetrabutylammonium fluoride in tetrahydrofuran, the 5′-*O* phosphonylated product **4** (dA^{phe}MP) was prepared by oxidation of the *H*-phosphonate monoesters thorough bis(trimethylsilyl) phosphite in CH_3CN solution in the presence of *N*,*O*-bis(trimethylsilyl)acetamide and (1*S*)-(+)-(8,8-dichlorocamphorsulfonyl)oxaziridine (S1). The mixture was partitioned between $CHCl_3$ and water. Using the triphosphorylation method (S2), the conversion of **4** to **5** (dA^{phe}TP) was accomplished using 1,1-carbonydiimidazole and tri-*n*-butylammonium pyrophosphate. This product was chromatographed on a C18 HPLC column using a triethylammonium acetate buffer (pH 7.0)/CH_3CN (80/20, v/v) to give **5**, with an overall yield of 11% from **1**. We verified that compound **5** was stable for at least several hours in the buffer at neutral pH.

REFERENCES

S1. Hoard,D.E., and Ott,D.G. (1965) Conversion of mono- and oligodeoxyribonucleotides to 5′-triphosphates. J. Am. Chem. Soc., 87, 1795-1788.

S2. Wada,T., Mochizuki,A., Sato,Y., and Sekine,M. (1998) A convenient method for phosphorylation involving a facile oxidation of *H*-phosphonate monoesters via bis(trimethylsilyl) phosphites. Tetrahedron Lett., 39, 7123-7126.

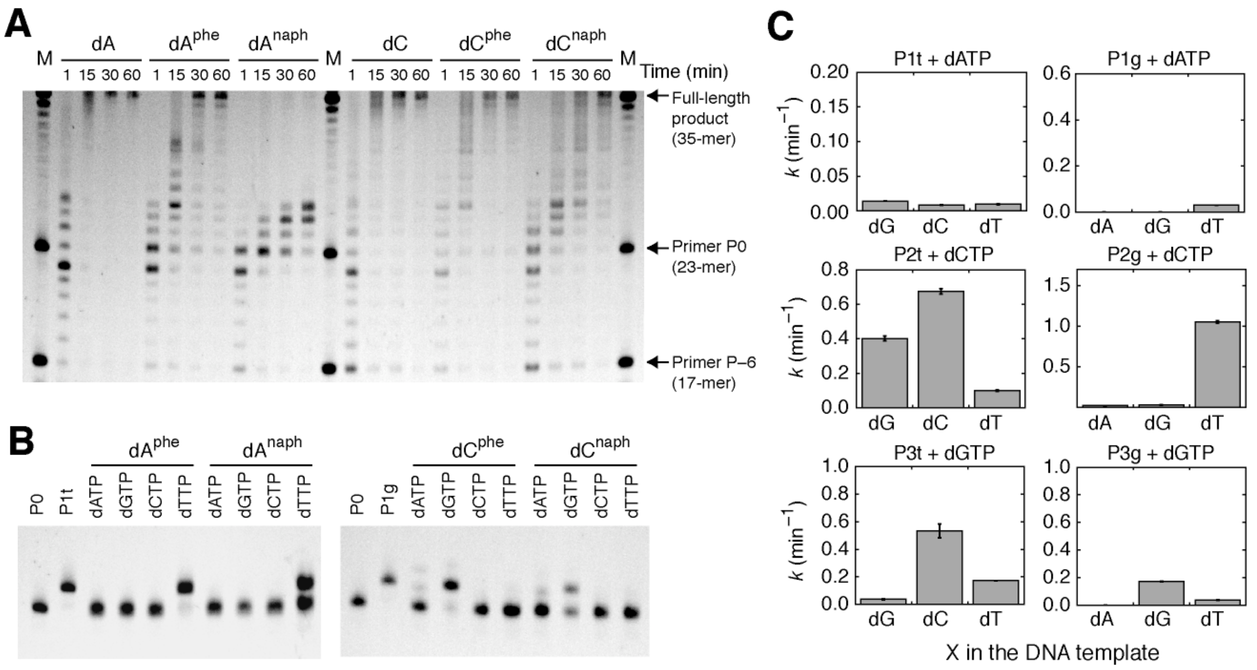

Figure S5. A. PAGE for the primer extension by the Klenow fragment using primer P–6 and the DNA templates presented in Figure 6A. The reaction was performed with a dNTP mix for 1, 5, 15, or 60 min, and M is the marker lane showing the 17-mer, 24-mer, and 35-mer lengths. B. PAGE demonstrating the selectivity of dNTP incorporation using primer P0 and T7 DNA polymerase. The reaction was performed with 120 µM dNTP for 10 min. C. Comparisons of the primer extension rates by the Klenow fragment using the template and primer strands forming a non-Watson-Crick base pair at the X position.

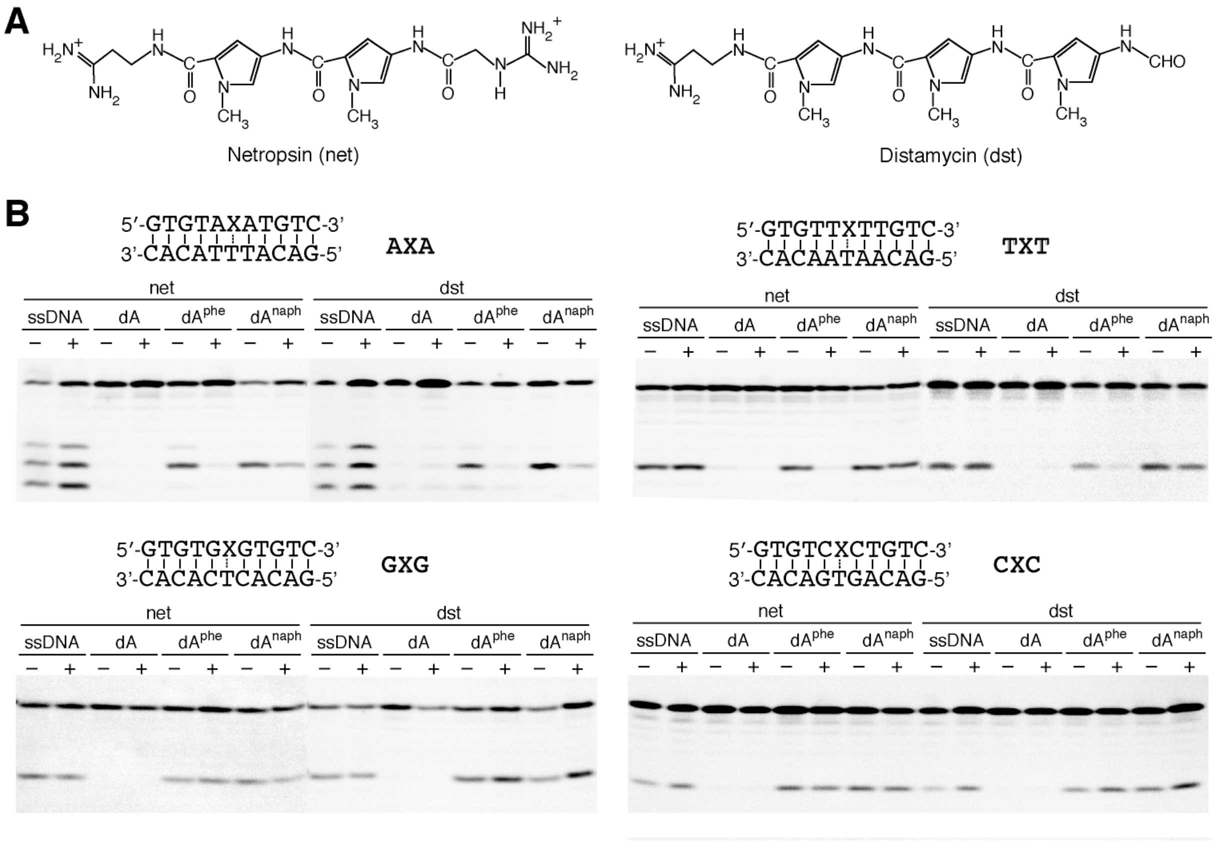

Figure S6. A. Chemical structures of netropsin and distamycin. B. PAGE for $KMnO_4$ oxidation of the DNA duplexes containing dA, dA^{phe}, or dA^{naph} at the X position in the absence (–) and presence (+) of netropsin or distamycin.
